# Potential of Platinum Standard Reference Genomes to Exploit Natural Variation in the Wild Relatives of Rice

**DOI:** 10.3389/fpls.2020.579980

**Published:** 2020-09-23

**Authors:** Saule Mussurova, Noor Al-Bader, Andrea Zuccolo, Rod A. Wing

**Affiliations:** ^1^ Center for Desert Agriculture, Biological and Environmental Sciences Division, King Abdullah University of Science and Technology, Thuwal, Saudi Arabia; ^2^ Institute of Life Sciences, Scuola Superiore Sant’Anna, Pisa, Italy; ^3^ School of Plant Sciences, Arizona Genomics Institute, University of Arizona, Tucson, AZ, United States

**Keywords:** neodomestication, rice wild relatives, platinum reference genomes, *Oryza coarctata*, *Oryza*

## Abstract

As the world’s population expands from 7.6 billion to 10 billion over the next 30 years, scientists and farmers across the globe must explore every angle necessary to provide a safe, stable and sustainable food supply for generations to come. Rice, and its wild relatives in the genus *Oryza*, will play a significant role in helping to solve this 10 billion people question due to its place as a staple food for billions. The genus *Oryza* is composed of 27 species that span 15 million years of evolutionary diversification and have been shown to contain a plethora of untapped adaptive traits, e.g., biotic and abiotic resistances, which can be used to improve cultivated rice. Such traits can be introduced into cultivated rice, in some cases by conventional crossing, and others *via* genetic transformation and gene editing methods. In cases where traits are too complex to easily transfer to cultivated rice [e.g., quantitative trait loci (QTL)], an alternative strategy is to domesticate the wild relative that already contains the desired adaptive traits – i.e., “neodomestication”. To utilize the *Oryza* genus for crop improvement and neodomestication, we first need a set of genomic resources that can be used to efficiently identify, capture, and guide molecular crop improvement. Here, we introduce the concept of platinum standard reference genome sequences (PSRefSeq) – a new standard by which contiguous near-gap free reference genomes can now be produced. By having a set of PSRefSeqs for every *Oryza* species we set a new bar for how crop wild relatives can be integrated into crop improvement programs.

## Introduction

As the world population grows and climate change dictates new conditions for agriculture worldwide, finding new solutions to ensure a sustainable food supply is crucial. Rice is the staple food in the regions of the world with the largest projected population growth. Moreover, these regions and their available arable land are expected to be most negatively affected by climate change in the future (Milovanovic and Smutka, 2017). Cultivated rice, as for most modern crops, has undergone domestication and genetic bottlenecks over millennia, giving this crop limited genetic potential to adapt to rapid environmental changes. In contrast, the wild relatives (WRs) of rice have been shown to possess the genetic potential to enhance yield, nutrient content, and resistance to several environmental stressors (Brar and Khush, 2018).

For decades, rice breeders have utilized genetic traits from the WRs of the genus *Oryza*, first *via* conventional crossing and now more recently using plant transformation and gene editing tools, to produce new, more resilient varieties of cultivated rice (Brar and Khush, 2018). Although these WRs contain novel alleles and variations with the potential to improve cultivated rice, issues such as hybrid-viability, -sterility, and -weakness hinder their introgression into cultivated rice, especially from the more distant relatives (Nadir et al., 2018). However, a toolbox of high-quality WR genome assemblies and gene-editing tools could help address these issues by providing technology for the precise transfer of newly discovered and advantageous wild alleles and genes into cultivated backgrounds.

Gene editing would also enable researchers to leverage these WRs with adaptive traits through neodomestication, i.e., the domestication of plants that have not previously been used for agriculture (Zsögön et al., 2018). As most traits in domesticated ideotypes arose from loss-of-function mutations, inducing such mutations in the orthologs of domestication-related genes in the WRs could result in a neodomesticated plant that maintains its desired adaptive trait(s) (e.g., abiotic and biotic resistance) (Doebley et al., 2006; Fernie and Yan, 2019). Though very far from a trivial task, neodomestication of the WRs of rice could potentially solve the challenge of feeding the planet’s rapidly growing population in the face of climate change. Neodomestication untangles the relationships between plant architecture, physiological mechanisms, and cellular processes that produce the complex and polygenic traits necessary to provide resistance or tolerance to environmental stressors.

Thus, in this review, we will discuss the following topics:

1. The untapped pool of genetic diversity buried within the genomes of the wild relatives of rice
2. The use of high-quality near gap-free reference genome sequences to aid in crop improvement
3. Barriers to genetic introgression and alternative strategies to introgression *via* neodomestication

## Genetic Diversity of the Wild Relatives of Rice

The *Oryza* genus contains 11 different genome types across 27 species, both diploid and polyploid, with a 3.6-fold difference in genome sizes (from around 300 Mb in the diploid FF genome of *O. brachyantha* to 1,283 Mb in the HHJJ polyploid genome of *O. ridleyi*, with the AA genome of cultivated rice falling in the 400Mb range). There are four species complexes within the *Oryza* genus: (1) *O. sativa*, (2) *O. officinalis*, (3) *O. ridleyi*, and (4) *O. meyeriana* (Vaughan, 1989). The WRs *O. rufipogon* and *O. barthii* have been identified as the progenitors of the domesticated rice species, *O. sativa* and *O. glaberrima*, respectively (Fuller et al., 2010; Wang et al., 2014; Chen et al., 2019). According to the classifications of gene pools initially defined by Harlan and de Wet, 1971, species in the *O. sativa* complex fall within the primary gene pool (Nadir et al., 2018). Despite reproductive barriers such as low cross-fertility, low F1 seed germinability and F2 hybrid weakness, common introgression within the *O.sativa* complex was observed and gene transfer requires traditional breeding methods (Jones et al., 1997; Jena, 2010; Zheng and Ge, 2010; Pusadee et al., 2016; Wang et al., 2017; Nadir et al., 2018). The secondary gene pool is represented by the *officinalis* complex and *O. brachyantha*, which is defined with regards to the primary gene pool as cross-incompatible with non-homologous chromosome pairing and require special techniques such as embryo rescue to achieve gene transfer (Jena, 2010). The tertiary gene pool complexes include the *O*. *meyeriana*, *O. ridleyi*, and unclassified *O. schlecteri* and *O. coarctata* species, which are highly cross-incompatible with *O.sativa*, producing anomalous, sterile or lethal hybrids (Jena, 2010; Brar and Khush, 2018). Taking into account their evolutionary history and known polyploidization events (both ancient and recent) (Ge et al., 1999; Lu et al., 2009), the WRs across the genus have successfully adapted to a wide range of habitats, which is reflected in their genomic content and associated phenotypes. A subset of these adaptive traits have been utilized for crop improvement, particularly the genes that control major biotic and abiotic stress resistances as well as yield-enhancement (Zhang K. et al., 2019).

The WRs of rice show a remarkable adaptive plasticity to a diverse set of habitats, often with extreme or suboptimal conditions (such as lack of freshwater, high temperatures, and flooding). This adaptivity has resulted in a range of beneficial traits that have been selected to “fine-tune” existing accessions of cultivated rice, which are crucial for adapting to changes in environmental conditions (**Table 1A**). For example, the first disease resistance gene cloned in rice was *Xa21* from the AA genome WR - *O. longistaminata* (Song et al., 1995). This gene confers resistance to rice blast, a devastating disease of rice. Another gene, *Bph18*, which provides resistance to the brown planthopper, was cloned from a more distant EE genome WR – *O. australiensis* (Ji et al., 2016). Both of these resistance genes have been introduced into *O*. *sativa* through marker-assisted selection to produce at least 13 resistant varieties grown in India, Philippines, China, and Korea (Sanchez et al., 2013). Abiotic traits such as salt stress tolerance in *O. coarctata*, heat tolerance *via* evening flowering in *O. australiensis*, early morning flowering (EMF) during the cooler morning hours in *O. officinalis*, as well as genes involved in low temperature adaptation have been identified in *O. rufipogon* are also strong candidates for introgression into cultivated rice (Zeigler et al, 2014; Bheemanahalli et al., 2017; Biswal et al., 2019).

**Table 1A:** Wild Species in the genus *Oryza* and available resources.

Species	2n	Genome Type	Distribution	Abiotic Traits of Interest	Ref	Biotic Traits of Interest	Ref
***Oryza sativa* complex**							
*O. rufipogon* sensu lato	24	AA	Asia, Oceania	Iron, acid, drought, submergence and aluminum tolerance	2,4	Resistance to BB, SB, tungro, and YSB	2, 3,5
*O. nivara*	24	AA		Drought Tolerance	4	Resistance to GSV and BB	2,3,5
*O. glaberrima* Steud.	24	AA	(cultivated)	Transpiration efficiency, heat, iron, acid, aluminum, salt, and drought tolerance	1,4	Resistance to BB and nematodes	5
*O. barthii* A. Chev.	24	AA	Africa	Heat and drought tolerance	1	Resistance to BB	5
*O. glumaepatula* Steud.	24	AA	Central/South America	Submergence Tolerance	4		
*O. meridionalis* Ng	24	AA	Oceania	Transpiration efficiency, heat tolerance	1,4		
*O. longistaminata* Chev. et Roehr	24	AA	Africa	Transpiration efficiency, heat and drought tolerance, and temperature plasticity	1	Resistance to GSV, BB, and YSB	2,3,5
***O. officinalis* complex**		multiple					
*O. punctata* Wall ex Watt, diplo.	24	BB	Africa	Drought tolerance and temperature plasticity	1	Resistance to BPH and ZLH	3
*O. schweinfurthiana* Wall ex Watt, tetra.	48	BBCC	Africa	Drought tolerance and temperature plasticity	1	Resistance to BPH and ZLH	3
*O. minuta* J.S. Presl ex C.B. Presl.	48	BBCC	Asia, Oceania			Resistance to BPH, GSV, BB, SB, and blast	2,3,5
*O. officinalis* Wall ex Watt	24	CC	Asia, Oceania	Drought and heat tolerance, and moisture plasticity	1,4	Resistance to BPH, GSV, BB, GLH, WPH, and blast	2,3,5
*O. rhizomatis* D.A. Vaughan	24	CC	Sri Lanka	Drought and submergence tolerance	4	Resistance to BB	5
*O. eichingeri* Peter	24	CC	Africa, Sri Lanka	Submergence tolerance	4	Resistance to BPH, WBPH, and GLH	3
*O. latifolia* Desv.	48	CCDD	Central/South America	Flooding tolerance, moisture plasticity	1	Resistance to GSV, BB, and BPH	2,3
*O. alta* Swallen	48	CCDD	Central/South America			Resistance to stem borer	3
*O. grandiglumis* (Doell.) Prod	48	CCDD	South America	Flooding and submergence tolerance	1,4		
*O. australiensis* Domin	24	EE	Australia	Transpiration efficiency, temperature and drought tolerance	1	Resistance to BPH, GSV, BB, and blast	2,3
***O. ridleyi* complex**		HHJJ					
*O. ridleyi* Hook	48	HHJJ	Asia, Oceania	Low radiation and moisture plasticity, and flooding tolerance	1	Resistance to blast, BB and stem borer	2,3
							
*O. longiglumis* Jansen	48	HHJJ	New Guinea			Resistance to BPH, BB and blast	2,3
***O. granulata* complex**		GG					
*O. granulata* Nees et Arn ex Watt	24	GG	Asia, Oceania	Cold tolerance, low radiation, temperature, moisture plasticity, and aerobic soil adaptation	1,3		
*O. meyeriana* (Zoll. et Mor. ex Steud.) Baill	24	GG	Asia, Oceania		1		
**Others**		multiple					
*O. brachyantha* Chev. et Roehr.	24	FF	Africa			Resistance to GSV, BB and yellow stem borer	2,3
*O. coarctata*	48	KKLL	Asia	Salt Tolerance	3,4		
O. *schlechteri* Pilger	48	HHKK	New Guinea	Flooding Tolerance	1		

BB, Bacterial blight; RYMV, ice yellow mottle virus; BPH, brown planthopper; WPH, whitebacked planthopper; GSV, grassy stunt virus; YSB, yellow stem borer; ZLH, zigzag leafhopper; GLH, Green leafhopper; 1 – Atwell et al., 2014; 2 – Toriyama, 2005; 3 – Jena, 2010; 4 – Menguer et al., 2017; 5-Vikal et al., 2007.

The WRs of rice have also been used as a source of other agronomic traits, such as yield. For example, *O. rufipogon*, the closest WR of Asian cultivated rice, was found to contain yield-enhancing QTL (*yld1* and *yld2*). These QTL were found to be associated with increased yield (18% and 17%) relative to the high-yielding Chinese hybrid rice V64 (Xiao et al., 1996). Subsequent studies have identified other QTL in *O. rufipogon* linked to yield component traits, including increased grain per panicle (e.g., *gpp3.1* identified in crosses with *O. rufipogon* and Jefferson which is a US japonica cultivar), and increased grain weight (e.g., *gw1.1* and *gw1.2* in crosses with Brazil Caiapo japonica cultivar) (Moncada et al., 2001; Thomson et al., 2003). Besides yield-component traits, a knowledge gap exists regarding WRs that may have superior nutritional and/or taste qualities. For example, seed from the KKLL genome WR *O. coarctata* is known for its sweet palatable taste and is consumed as a local delicacy in Bangladesh (Kabir and Humayun, 2012). Overall, such traits could potentially be utilized in cultivated rice to address the nutritional needs and flavor preferences of a growing population.

Beyond the association of traits to genes-of-interest, polyploidy in crops often confers greater genome plasticity and adaptation. The polyploid WRs of rice are likely to be more adaptive than their extinct diploid progenitors and may exhibit higher adaptability to extreme habitats, different climates, soil types, drought/submergence conditions, and biotic stresses (Van de Peer et al., 2017). In rice, hormone responses and differential expression of stress-response genes due to polyploidy confer autotetraploid rice with better drought-tolerance characteristics than diploid rice. Autotetraploid *O. sativa* (produced by colchicine-treatment to cause genome doubling) was less affected under severe drought stress, with less effect on net photosynthetic rates and peroxidation levels of its cell membranes demonstrated higher activity of the enzymes (superoxide dismutase, peroxidase, catalase) implicated in decreasing the amount of reactive oxygen species, which limited membrane lipid peroxidation (Yang et al., 2014). The authors hypothesized that the gene dosage effects due to polyploidy are the reason for drought tolerance in autotetraploid *O. sativa*. Similarly, polyploidy was found to confer rice growth and survival advantages under salt stress. When exposed to salt stress, four pairs of polyploid accessions, created from diploid progenitors, presented higher salt tolerance *via* dry weight, chlorophyll a/b content, and mortality rates of the tetraploids (Jiang et al., 2013). In addition, root ultrastructure imaging revealed decreased membrane damage and increased stability of nuclei and membrane organelles in the roots of tetraploid rice compared to diploid rice under salt stress (Tu et al., 2014). As the salt-induced production of reactive oxygen species affects membrane integrity *via* lipid/protein peroxidation, it is hypothesized that increased stability of membranes, nuclei and membrane organelles is an indication of normal metabolism under salt stress in roots of the tetraploid rice (Tu et al., 2014). Previously, tetraploid rootstocks in citrus have been demonstrated to be more tolerant to salt stress as compared to the corresponding diploid rootstocks (Saleh et al., 2008). These examples show that polyploidy can be advantageous due to potential dosage effects. However, a better understanding of genome content and structure is required, besides the trait-associated genes and QTL, to identify advantageous traits in the WRs for their introgression into cultivated backgrounds.

## Use of High-Quality Reference Genomes to Aid in Crop Improvement

For crop improvement of the genus *Oryza*, breeders require access to a comprehensive set of genomic tools that can bridge the 27 species and 11 genome types that *Oryza* has accumulated over 15 million years of evolutionary history. The International *Oryza* Map Alignment Project (I-*O*MAP), established in 2003, set out to create a genus-level comparative genomics platform for the interrogation of the genus *Oryza* and provide information on rice-related genome evolution and organization, comparative genomics, physiology, biochemistry, and crop improvement. I-*O*MAP began with the generation of a set of publicly available deep-coverage BAC libraries and manually edited physical maps for cultivated rice and WRs used in active breeding programs (Jacquemin et al., 2013). These resources led to many early discoveries on the genome biology of *Oryza*, and grasses in general, and facilitated the generation of several draft genome assemblies, including that of African rice (Wang et al., 2014; Stein et al., 2018).

Recently, I-*O*MAP generated a set of 15 PSRefSeqs from one representative accession for each of the 15 sub-populations of *O. sativa*, based on the 3,000 rice genome (3K-RG) dataset, to be used as a reference guide to characterize all standing natural variation within Asian cultivated rice (Zhou et al., 2020). A PSRefSeq is defined as a high-quality near gap-free chromosome-level reference genome validated with optical maps. The *O. sativa* PSRefSeq dataset is composed of 12 newly sequenced genomes (Zhou et al., 2020), and 3 previously published genomes for Minghui 63 (MH63), Zhenshan 97 (ZS97) and N22 (Zhang et al., 2016a; Zhang et al., 2016b; Stein et al., 2018). The average number of contigs for these 15 assemblies is 113 contigs (i.e., 912, 237, and 181 for N22, MH63, and ZS97, respectively, and an average of 30 contigs for the remaining 12). The average number of gaps across the 15 assemblies is 46, with 8 out of 15 having less than 10 gaps (Zhou et al., 2020).

For ZS97 and MH63, genome completeness was estimated at ~92.7% (ZS97), ~94.8% (MH63) using the CEGMA pipeline with a core set of 248 eukaryotic genes (Parra et al., 2009). BUSCO evaluations for the remaining 13 genomes, which interrogate a much larger core gene set (N=956 for N22 and N=1,427 for the 12 newly sequenced assemblies), averaged 98.6%, and demonstrates the high contiguity and completeness of the majority of the *O. sativa* PSRefSeq dataset (Simão et al., 2015). When compared with previously released rice genomes, the *O. sativa* PSRefSeq data set, described here, provides more contiguous and accurate sequence data which, thereby, will improve further downstream analysis such as TE (transposable element), centromere/telomere and gene annotations.

The *O. sativa* 15 PSRefSeq dataset, combined with the original IRGSP RefSeq (International Rice Genome Sequencing Project, 2005; Kawahara et al., 2013), presents a multiple reference pan-genome template for the primary gene pool of Asian cultivated rice for mapping resequencing data that can accurately characterize genetic variation at the subpopulation level (Zhou et al., 2020). Given that this PSRefSeq dataset is now publicly available, the next logical step is to produce PSRefSeqs that represent the secondary and tertiary gene pools of the genus *Oryza*, and when combined, will create an unprecedented pan-genome for the entire genus – i.e., a “pan-genus-genome” or *Oryza*-PGG. Currently, I-*O*MAP is producing a set of PSRefSeqs from the representatives of all 25 WRs and *O. glaberrima*, using a combination of long-read sequencing technologies and optical maps (Udall and Dawe, 2018), with a target release date of December 2020.

The *Oryza* PGG will provide the full range of short, medium, and long-range structural variations that exist across the genus. Moreover, the *Oryza* PGG will confirm previously discovered structural variants (SVs) and the presence/absence of variants (PAVs). Zhou et al. (2020) recently showed that the majority of PAVs in the subpopulations of *O. sativa* are comprised of transposable elements (TEs). The *Oryza* PGG will allow us to better annotate and understand TEs in the WRs of rice and shed light on the contribution of TEs to genome size variation and genome plasticity of adaptive traits. For instance, the TE content of *O. coarctata* was estimated to be the lowest among *Oryza* species, despite a predicted genome size of 665 Mb (Zuccolo et al., 2007; Mondal et al., 2018).

Analyses of the *Oryza* PGG will also reveal the vast majority of single gene and gene family content and evolution across the genus. Previously, Zhang L. et al. (2019) leveraged the genomic data available for 13 *Oryza* species (domesticated and wild) to study the formation of *de novo* protein-coding genes. They identified 175 candidate *de novo* genes and estimated the rate of *de novo* gene origination as 51.5 *de novo* genes per MYA. *De novo* originated genes can provide novel biological functions and have previously been shown to play a key role in pathogen resistance in plants (Chen et al., 2013). For instance, *OsDR10* is an *Oryza*-lineage specific *de novo* originated gene that confers broad-spectrum bacterial blight resistance when suppressed in rice (Xiao et al., 2009). Currently, *OsDR10* is characterized as an “orphan” gene and there is currently little information on its evolution in the *Oryza* lineage. Once the *Oryza* PGG becomes available, the evolutionary mechanisms contributing to the generation of *OsDR10* may be revealed and thus, provide insights into new gene evolution across the genus.

Finally, PSRefSeqs of each species will also aid conservation efforts by serving as “platinum standard” templates for characterizing the diversity of seed bank materials. This information will guide the management of genetic resources through *in-situ* conservation efforts to capture the genetic diversity in the wild or *ex-situ* collections of the newly identified wild populations (Brozynska et al., 2016). Some of the WRs in the genus are endangered or, as in the case of *O. neocaledonica* (Molla et al., 2018) and *O.schlecteri* absent from known genebank collections (Germplasm Resources Information Network, n.d.). From an evolutionary perspective, gained or lost SVs and copy number variations (CNVs) can be indicative of local adaptation of wild populations to unique habitats. The information gained from PSRefSeqs will help in the long-term maintenance of wild populations in their native habitats.

## Barriers to Genetic Introgression and Alternative Strategies to Introgression *via* Neodomestication of WR Rice Species

For the successful introgression of desired traits from the WRs of rice, incompatibility barriers between the secondary, tertiary, and primary *Oryza* gene pools must be overcome (Harlan and de Wet, 1971; Vincent et al., 2013). The introgression of desirable traits within the AA genomes is relatively straightforward compared to the introgression of traits from more distant complexes. For instance, crossing WRs from the secondary/tertiary gene pools is more laborious and can require embryo rescue and chromosome doubling to produce interspecific hybrids and alien introgression lines (AILs). For the closely related *O. officinalis* complex species, monosomic alien addition lines (MAALs) and interspecific hybrids, with *O*. *sativa* as the recurrent parent, have been successfully produced to carry the following biotic stress-resistance traits: brown planthopper resistance, whitebacked planthopper, and bacterial blight resistances from *O. officinalis*, *O. minuta*, and *O. latifolia*, respectively (Brar and Khush, 2018).

Despite the successes achieved by introgression from the *O. officinalis* complex, crossing *O. sativa* with more distant WRs is difficult and time-consuming, and can result in progeny depression or complete progeny sterility. Moreover, linkage drag of loci, related to negative traits such as low yield, and shattering can also occur (Xiao et al., 1998). For the *meyeriana* complex, breeding efforts resulted in 40 derived lines of *O. sativa* x *O. granulata*, some of which were found to contain partial alien chromosome introgressions from two out of six *O. granulata* chromosomes analyzed. However, no favorable traits (such as cold and low radiation tolerances) were found to be introgressed from *O. granulata* (Brar et al., 1996). Similarly, attempts to produce hybrids from *O. sativa* x *O. ridleyi* and *O. sativa* x *O. coarctata* crosses failed due to necrosis in the progeny or progeny sterility, respectively, thereby demonstrating the difficulty of hybrid formation between distant WRs of rice with *O. sativa* (Brar and Khush, 2018).

“Neodomestication” represents an alternative strategy to conventional trait introgression from a WR into a domesticated species. This method preserves adaptations to biotic/abiotic stresses while exhibiting the traits of a domesticated crop (e.g., loss of shattering, erect growth, larger seeds). For example, homologues of genes involved in the domestication of rice in the WRs could be targeted for neodomestication to produce a domesticated ideotype in a WR. Shapter et al. (2013) presented an early example of an accelerated neodomestication process using random EMS mutagenesis to initiate the domestication of weeping rice grass **(** *Microlaena stipoides*), which resulted in the generation of two non-shattering mutant plants in the orthologs of *qSH1* and *sh4*. Now, with gene editing technology such as CRISPR-Cas9 (Bortesi and Fischer, 2015), it is possible to precisely edit homologues of well-studied domestication genes in crop WRs, such as those listed in **Table 1B**, to activate genes, modify alleles, introduce targeted base substitutions, delete large genomic segments, or introduce complete genes previously unavailable in a given species (Fernie and Yan, 2019). Besides targeting orthologues of domestication genes for neodomestication of WR *Oryza* candidates, CRISPR/Cas9 gene editing could be used to introduce WR candidate genes implicated in favourable traits such as abiotic stress tolerances into the cultivated rice background by overcoming the reproductive *Oryza* gene pool barriers (Harlan and de Wet, 1971).

**Table 1B:** Domestication genes in *Oryza sativa*.

Gene Name	RAP Gene ID	*Oryza* specie	Protein category	Domestication Trait	Ref
*SH4*	Os04g0670900	*O.sativa indica* x *O.nivara*	Myb3 transcription factor	Loss of seed shattering: the absence of abscission layer formation	Li et al., 2006
*qSH1*	Os01g0848400	*O.sativa* Nipponbare x *O.sativa* Kasalath	BEL1-type transcription factor	Loss of seed shattering: the absence of abscission layer formation	Konishi et al., 2006
*PROG1*	Os07g0153600	*O. rufipogon* x *O.sativa*	Cys(2)-His(2) zinc-finger protein	Erect growth and high yield	Tan et al., 2008; Wu et al., 2018
*OsLG1*	Os04g0656500	*O. rufipogon* x *O.sativa*	SBP-domain transcription factor	Panicle shape: closed panicle	Ishii et al., 2013
*Rc*	Os07g0211500	*O. rufipogon* x *O.sativa*	bHLH-protein	Seed pericarp color: white pericarp	Sweeney et al., 2006
*Bh4*	Os04g0460000	*O.sativa, O.rufipogon, O.nivara, O.glaberrima, O.barthii*	Amino acid transporter	Seed hull color: straw-white seed hull	Zhu et al., 2011; Vigueira et al., 2013
*LABA1*	Os04g0518800	*O. rufipogon* x *O.sativa*	Cytokinin-Activating Enzyme	Awn morphology: short, barbless awns	Hua et al., 2015
*Ehd4*	Os03g0112700	*O.sativa* Kita-ake	CCCH-type zinc finger protein	Flowering time: photoperiodic control of flowering	Gao et al., 2013
*Hd6*	Os03g0762000	*O.sativa* Nipponbare x *O.sativa* Kasalath	CK2alpha protein kinase subunit	Flowering time: delay of flowering under long-day conditions	Yamamoto et al., 2000
*Hd3a*	Os06g0157700	*O.sativa* Nipponbare x *O.sativa* Kasalath	Rice ortholog of *Arabidopsis* FT (mobile flowering signal)	Flowering time: transition to flowering under short-day conditions	Yamamoto et al., 1998; Komiya et al., 2008

As a proof-of-concept study for neodomestication using gene editing, Zsögön et al. (2018) targeted six key domestication traits for loss of function in the wild tomato *S. pimpinellifolium* (i.e., *SP, SELF-PRUNING; O, OVATE; FW2.2, FRUIT WEIGHT; LYCOPENE BETA CYCLASE, FASCIATED, MULTIFLORA*). Four of the six genes (i.e., all except *FASCIATED* and *MULTIFLORA*) were successfully targeted and contained indel mutations, which resulted in a WR with a domesticated “phenotype”, i.e., an altered morphology, a three-fold increase in fruit size, a ten-fold increase in fruit number compared to the WR, and a 500% increase in lycopene accumulation in the fruit, relative to the cultivated *Solanum lycopersicum* (Zsögön et al., 2018).

Similarly to wild tomato, neodomestication of the WRs of rice is theoretically possible, albeit non-trivial. *O. coarctata* is an attractive candidate for neodomestication because it is the only halophyte in the *Oryza* genus and can thrive in salinity levels of up to 40 E.CedS m^−1^ (i.e., brackish water salinity levels) along the coastal regions from Pakistan to Myanmar (Bal and Dutt, 1986). In India, the habitat of this species is similar to that of mangrove forests where *O. coarctata* can be found submerged in saline water for up to 12 h a day due to lunar tides (Bal and Dutt, 1986; Mondal et al., 2018). For decades, rice breeders have tried to introgress the halophyte characteristics of *O. coarctata* into cultivated rice with limited or no success (Brar and Khush, 2018; Prusty et al., 2018). Thus, *O. coarctata* is an ideal candidate for neodomestication and, if successful, would allow farmers to grow rice on land that otherwise is unable to support conventional rice farming practices. A proposed methodology for the neodomestication of *O. coarctata* includes the targeting of orthologues of domestication genes in *O. coarctata* (**Table 1B**) with precise gene editing. An *O*. *coarctata* PSRefSeq will enable less off-target CRISPR/Cas9 enzyme effects (not attributed to the enzyme mode of action) as the contiguity and fidelity of the PSRefSeq allows for better precision for single guide RNA (sgRNA) selection (Biswal et al., 2019). Moreover, *O. coarctata*, unlike *S. pimpinellifolium*, is not a direct progenitor of the cultivated *O. sativa* and is a polyploid which, besides affecting the number of domestication-related genes, also translates to a vast difference in the genome structure, thereby making gene editing more challenging due to the potential linkage of domestication genes to non-desirable traits (i.e., linkage drag). Given the paucity of available genome assembly resources, a PSRefSeq for *O. coarctata* would play a vital role in the precise identification of the orthologues of domestication genes that could be targeted for neodomestication.

## Author Contributions

SM and NA-B contributed equally to this work and share co-first authorship. SM and NA-B wrote the manuscript. AZ contributed to writing the manuscript, editing and organizing the structure of the manuscript. RW reviewed and edited the manuscript. All authors contributed to the article and approved the submitted version.

## Funding

This work was supported by KAUST to RW.

## Conflict of Interest

The authors declare that the research was conducted in the absence of any commercial or financial relationships that could be construed as a potential conflict of interest.
